# Hydrogen-Rich Gas Production by Steam Reforming and Oxidative Steam Reforming of Methanol over La_0.6_Sr_0.4_CoO_3−δ_: Effects of Preparation, Operation Conditions, and Redox Cycles

**DOI:** 10.1021/acsaem.3c00778

**Published:** 2023-07-25

**Authors:** Miguel Morales, Miguel Ángel Laguna-Bercero, Emilio Jiménez-Piqué

**Affiliations:** † CIEFMA—Department of Materials Science and Engineering, EEBE—Campus Diagonal Besòs, Universitat Politècnica de Catalunya—BarcelonaTech, C/Eduard Maristany 16, 08019 Barcelona, Spain; ‡ Barcelona Research Center in Multiscale Science and Engineering, Universitat Politècnica de Catalunya—BarcelonaTech, C/Eduard Maristany 16, 08019 Barcelona, Spain; § Instituto de Nanociencia y Materiales de Aragón, INMA, CSIC, Universidad de Zaragoza, Pedro Cerbuna 12, 50009 Zaragoza, Spain

**Keywords:** methanol, hydrogen production, steam reforming, nanoparticles, perovskite, solid oxide fuel cells

## Abstract

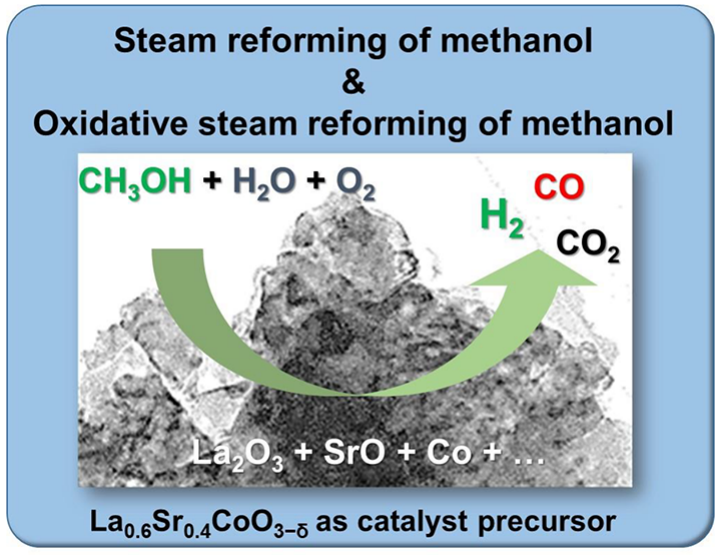

La_0.6_Sr_0.4_CoO_3−δ_ (LSC) perovskite, as a potential catalyst precursor for hydrogen (H_2_)-rich production by steam reforming of methanol (SRM) and oxidative steam reforming of methanol (OSRM), was investigated. For this purpose, LSC was synthesized by the citrate sol–gel method and characterized by complementary analytical techniques. The catalytic activity was studied for the as-prepared and prereduced LSC and compared with the undoped LaCoO_3−δ_ (LCO) at several feed gas compositions. Furthermore, the degradation and regeneration of LSC under repeated redox cycles were studied. The results evidenced that the increase in the water/methanol ratio under SRM, and the O_2_ addition under OSRM, increased the CO_2_ formation and decreased both the H_2_ selectivity and catalyst deactivation caused by carbon deposition. Methanol conversion of the prereduced LSC was significantly enhanced at a lower temperature than that of as-prepared LSC and undoped LCO. This was attributed to the performance of metallic cobalt nanoparticles highly dispersed under reducing atmospheres. The reoxidation program in repetitive redox cycles played a crucial role in the regeneration of catalysts, which could be regenerated to the initial perovskite structure under a specific thermal treatment, minimizing the degradation of the catalytic activity and surface.

## 1 Introduction

Catalytic steam reforming of methanol (SRM) presents a great potential to supply hydrogen (H_2_) for fuel cells.^1^ Although methanol (CH_3_OH) is highly toxic and miscible in water, it possesses several advantages, such as a high hydrogen-to-carbon ratio, liquid state at atmospheric conditions, and biodegradability.^2^ Compared to other fuels like hydrogen, synthesis gas, or methane, the hydrogen volumetric density in methanol is higher than those of either compressed fuel gas or liquid H_2_. It is easier to distribute with the existing infrastructure and safer to handle than compressed H_2_.^3,4^ Like other fuels, methanol can be a carbon-neutral renewable feedstock because it is easily obtained as a synthetic fuel from renewable sources.^5,6^ On the other hand, the operating temperatures for SRM (200–300 °C) are lower compared to other reforming processes such as ethanol steam reforming around 400 °C^7,8^ or methane steam reforming above 500 °C.^9^ The SRM process is mainly described by three main reactions: direct SRM (eq 1), methanol decomposition (MD) (eq 2), and reverse water–gas shift (RWGS) (eq 3).^10,11^
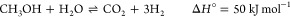
1
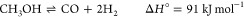
2
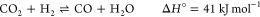
3

MD (eq 2) and RWGS (eq 3) occur simultaneously with SRM. Because the overall process is remarkably endothermic, it consumes so much energy that it has to be supplied from an external source. The addition of O_2_ to the SRM process is a common practice to combine the steam reforming and partial oxidation reactions that could approximate an autothermal oxidative steam reforming of methanol (OSRM; eq 4):
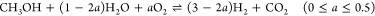
4

Autothermal SRM can be reached at around *a* = 0.1–0.2.^12^ Higher O_2_ compositions favor the formation of CO_2_ and H_2_O, as well as a strongly exothermic process, because partial oxidation becomes predominant.

Typically, the use of SRM includes the production of H_2_ for proton exchange membrane fuel cells (PEMFCs). However, the formation of CO, as a byproduct of reforming, is one of the drawbacks of SRM because CO concentrations above 10 ppm lead to catalyst poisoning of the PEMFC anode.^1^ Thus, the catalyst used in the SRM process must possess high catalytic activity at low temperatures, minimization of CO production, high hydrogen selectivity, and high stability. SRM may be effectively carried out over the commercially available catalyst based on either Cu or Pd supported typically on ZnO and Al_2_O_3_.^13^ Pd-based catalysts exhibit better thermal stability than Cu-based ones with a CO_2_ selectivity close to 100%.^14,15^ The optimal operating conditions of Cu/Zn/Al catalysts, mainly used in small hydrogen plants, are close to the H_2_O/CH_3_OH ratio of 1.3 and a temperature range of 250–300 °C.^16^

Alternatively, perovskite-type oxides, with the general formula ABO_3±δ_, where A represents rare earths (e.g., La) or alkali/alkaline-earth (e.g., Ca, Sr) metal cations and B depicts transition-metal cations (e.g., Co, Cu, and Fe), are promising highly active catalyst precursors to produce H_2_ from different reforming reactions of alcohols and hydrocarbons.^17,18^ Thus, their catalytic properties can be tailored for specific applications by partially substituting A and/or B site cations with suitable ones while preserving the perovskite crystal structure. Lanthanum cobaltite perovskites have been implemented as catalyst precursors for many oxidation and reduction reactions in heterogeneous catalysis.^18^ Among others, Cu- and Pd-doped perovskites of the La/SrCo_1–*x*–*y*_M_*x*_M_*y*_O_3±δ_ (M = Cu, Pd, Zn) type are especially interesting for SMR because the presence of Cu and Pd in the LaCoO_3_ structure increases O-ion mobility and enhances the catalytic activity of SMR. For instance, LaCo_0.7_Cu_0.3_O_3−δ_,^19^ LaCo_1–*x*–*y*_Pd_*x*_Zn_*y*_O_3±δ_,^20,21^ La_2_CuO_4_/CuO/ZnO/Al_2_O_3_,^22^ CuO/La_1–*x*_Ce_*x*_CrO_3_,^23^ and SrCo_1–*x*_Cu_*x*_O_3±δ_^24^ are good candidates for the SRM process because their metal oxides generated in reducing atmospheres can stabilize Cu and/or Pd, avoiding migration and sintering of particles. Nevertheless, these perovskite-type oxides perform poorly at relatively low temperatures compared to Cu or Pd supported on ZnO/Al_2_O_3_ because CH_3_OH conversion starts above 250–300 °C and the CO_2_ selectivity is between 50% and 70%. In this regard, these mixtures of H_2_ and CO, named synthesis gas (syngas), can be attractive for feeding solid oxide fuel cells (SOFCs).^25^ Compared to PEMFCs, SOFCs are much more flexible because they can effectively use different mixtures of H_2_, CO, and other gases as fuels. Therefore, lanthanum cobaltite perovskites may present a high potential use for SRM to supply SOFC applications, but unfortunately literature is very scarce. To the best of the authors’ knowledge, only LaCo_0.7_Cu_0.3_O_3−δ_,^19^ LaCo_1–*x*–*y*_Pd_*x*_Zn_*y*_O_3±δ_,^20^ and La_0.6_Sr_0.4_Co_1–*y*_Fe_*y*_O_3−δ_^26^ have reported high CH_3_OH conversions and H_2_ and CO_2_ selectivities of about 80–90% and 40–70%, respectively. The SRM performance significantly depended on the catalyst composition, enhanced by increasing Co/Fe ratio.^20^ The partial substitution of Co with both Pd and Zn considerably suppressed the decomposition reaction, thus increasing the CO_2_ selectivity.^26^ In addition, the SRM activity was also affected by the H_2_O/CH_3_OH ratio, yielding better CH_3_OH conversion and CO_2_ selectivity with excess H_2_O in the feed.^20^ The reaction mixture obtained higher catalytic activity when O_2_ was added.^19^

Given the works above, Sr-substituted lanthanum–cobalt oxides (La_1–*x*_Sr_*x*_CoO_3−δ_) may be another interesting perovskite-type oxide for SRM and OSRM, particularly when a high H_2_/CO ratio is not required. Then, the absence of Pd in the catalysts could be an advantage in decreasing their price. Furthermore, the strontium oxide in LaXCoO_3_ (X = Mg, Ca, Sr, Ce) could enhance the activity and stability, such as for steam reforming of ethanol, showing a better catalytic performance than the other compositions.^27^ In this regard, La_1–*x*_Sr_*x*_CoO_3_ is known to be interesting because of its excellent performance for multiple catalytic applications: oxidation of CO, propane, and methane^28,29^ soot oxidation activity,^30^ toluene combustion,^31^ Li–O_2_ battery,^32^ and solar photocatalyst.^33^ Furthermore, La_1–*x*_Sr_*x*_CoO_3−δ_ (*x* = 0.2–0.5) is typically used, showing excellent performances as an oxygen electrode and a current collector for SOFCs^34−36^ and solid oxide electrolyzer cells (SOECs)^37−39^ and also as a cathode for single-chamber SOFCs.^40,41^ Among the studied compositions of La_1–*x*_Sr_*x*_CoO_3−δ_ for catalytic applications, La_0.6_Sr_0.4_CoO_3−δ_ (LSC) showed the best catalytic activity, attributed to the highest oxygen chemisorption and desorption capacity.^28−30^ In this regard, Morales et al.^42−44^ reported the performance and stability of La_1–*x*_Sr_*x*_CoO_3−δ_ (*x* = 0.4 and 0.5) perovskites, as a catalyst precursor, for the production of H_2_ and syngas by the partial oxidation of methane and steam reforming of ethanol. The results evidenced a remarkable catalytic activity due to the stability of Co particles, which were highly dispersed in their reduced state. Similarly, Chen et al.^45^ recently reported a series of A-site Sr-doped La_1–*x*_Sr_*x*_CeO_3−δ_ (*x* = 0.2, 0.4, 0.6, 0.8) perovskite catalysts for H_2_ production by SRM, showing that La_0.6_Sr_0.4_CeO_3−δ_ exhibited the best performance at optimal reaction conditions. In summary, Sr-substituted lanthanum–cobalt oxide perovskites may be potential catalytic precursors for the SRM and OSRM processes to supply H_2_-rich gas for SOFC applications. However, few studies reported the SRM and OSRM over LSC perovskites, and information on how the preparation, operation conditions, and degradation under redox cycles affected the catalytic activity is still scarce. Meanwhile, these issues also have a great interest in the SOFC development using LSC perovskites because their degradation is primarily due to Sr segregation on the electrode surfaces at operating temperatures higher than 500 °C.^46,47^ Recently, several strategies to control Sr segregation have been reported on the modification of surfaces with more and less reducible cations, the control and change of the oxygen partial pressure and applied voltage, the substitution of isovalent A- or B-site cations with different ionic radii, or the lowering of the operating temperature to deactivate Sr segregation thermodynamically and kinetically, among others.^48−50^ Despite many efforts, completely effective practical solutions have not yet been proposed to inhibit Sr segregation, and, therefore, the use of thermal treatments and HCl etching to remove the Sr-rich particles at the surface of the used LSC have been proposed.^50^

Here, we propose to study the LSC under multiple SRM and OSRM conditions because understanding the preparation, operation conditions, and redox cycles of SRM and OSRM perovskites is critical for finding new strategies, optimizing their performance, and enabling their practical application in catalysis but also in other systems such as SOECs for renewable energy conversion and storage. In the present work, LSC was studied as a precursor catalyst for SRM and OSRM. For this purpose, LSC was synthesized by the sol–gel citrate method. The prepared material was deeply characterized by different complementary analysis techniques, and, subsequently, the catalytic activity toward SRM and OSRM was investigated. Particular attention was paid to studying the effect of LSC’s reductive thermal pretreatment and feed gas composition on the catalytic performance. Furthermore, the degradation and regeneration of LSC under several reaction conditions and repeated redox cycles were investigated.

## 2 Experimental Procedure

### 2.1 Catalyst Preparation

La_0.6_Sr_0.4_CoO_3−δ_ (LSC) perovskite was synthesized by the sol–gel citrate method. The detailed synthesis procedure was described elsewhere.^42^ Metal precursors La(NO_3_)_3_·6H_2_O (Alfa Aesar, 99.9%), Sr(NO_3_)_2_ (Alfa Aesar, 99.9%), and Co(NO_3_)_2_·6H_2_O (Alfa Aesar, 99.9%) were weighed in stoichiometric amounts and dissolved in deionized water. Subsequently, citric acid and ethylenediaminetetraacetic acid were added to the metal ion solution. The aqueous solution was slowly evaporated under stirring at 75 °C until a gel was obtained. The resulting gel was dried at 110 °C, homogenized in an agate mortar, and calcined at 500 °C to promote decomposition of the organic fraction. Finally, the precursor was calcined at 900 °C in synthetic air for 12 h and at 300 °C in pure O_2_ for 72 h.

### 2.2 Catalyst Characterization

The catalyst was characterized in different oxidation states: as-prepared LSC, after a reductive thermal treatment in 5% H_2_/Ar at 650 °C for 1 h, after SRM and OSRM tests, and after a specific thermal treatment of regeneration. To investigate *in situ* regeneration of the LSC precursor under the reaction conditions, redox cyclic tests were carried out, reoxidizing the catalyst by decreasing the temperature from reaction temperature (600 °C) to room temperature (RT) in several cooling programs: a quenching in N_2_, a quenching under SRM and OSRM, and a slow cooling under SRM, OSRM, and air. Regeneration experiments of the LSC precursor were also carried out under synthetic air at 900 °C for 12 h and subsequently at 300 °C for 72 h, similar to that described previously.^43,44^

The specific surface area of the catalysts was determined by the Brunauer–Emmett–Teller method using a Micromeritics model Tristar 3000, measuring the nitrogen adsorption at 77 K. Temperature-programmed reduction (TPR) experiments were performed in a reactor equipped with a thermal conductivity detector (TCD). A gas mixture of 5% H_2_/Ar (30 mL min^–1^) was used to reduce 50 mg of LSC by heating from 100 to 800 °C at 10 °C min^–1^. Phase analysis of the synthesized and thermally treated powders was performed by X-ray diffraction (XRD). The XRD patterns were obtained by using a Siemens 2000 diffractometer with Cu Kα radiation (40 kV, 40 mA, λ = 0.154 nm). The XRD patterns were collected in the range 2θ = 20–80°, and the crystalline phases were identified using the JCPDS database. The crystallite sizes were estimated through the line broadening of the XRD peaks using the Scherrer equation.^51^ The surface properties related to the chemical states and surface compositions of La, Sr, Co, and O in the LSC samples were determined by X-ray photoelectron spectroscopy (XPS). XPS analyses were conducted in an ultrahigh-vacuum multichamber system by SPECS with a PHOIBOS 150 EP hemispherical energy analyzer and an MCD-9 detector XR-50. It possesses an X-ray source with a twin anode (Al and Mg) and a high-pressure and high-temperature chamber for gas treatments of the samples. The samples were compensated for charging with a low-energy electron beam, and the peak of C 1s (binding energy = 284.4 eV) was used to correct for sample charging effects.^52^*SpecsLab Prodigy*, an experiment control software package, was used for data acquisition and *CasaXPS* for spectral analysis. The catalyst microstructures were observed by transmission electron microscopy (TEM; Titan G2 60-300, USA) and scanning electron microscopy (SEM; Carl Zeiss Merlin, Germany) equipped with energy-dispersive spectroscopy (EDS; Oxford Instruments INCA-350 system, United Kingdom). The samples were coated with carbon to minimize the electrical charge on the surface.

### 2.3 Catalytic Tests

SRM and OSRM experiments were carried out in a fixed-bed quartz tubular reactor (5 mm inner diameter) at atmospheric pressure. The catalytic tests were performed using 50 mg of LSC packed on a bed of quartz wool in the reactor. The reactor was kept in a horizontal tubular furnace. Two K-type thermocouples were used: one outside the reactor to control the furnace temperature using a Eurotherm PID controller and another in contact with the catalyst to measure the bed temperature. Before catalyst tests, several blanks (without catalyst) were analyzed to confirm the reactor’s absence of direct oxidation reactions. Before the SRM reaction, the catalyst precursor was reduced at 650 °C for 1 h in 5 vol % H_2_/Ar (30 mL min^–1^) and then cooled under N_2_ (30 mL min^–1^) to the initial testing temperature of 150 °C. In addition, several tests were performed with the as-prepared catalyst (without reductive pretreatment) to study the effect of the previous oxidation state of the catalyst on the catalytic activity. Afterward, the reaction mixture was introduced to the reactor to analyze the impact of the temperature, varying the feed composition of the O_2_/H_2_O/CH_3_OH molar ratio on the methanol conversion. Finally, the selectivities of the products were investigated under different SRM and OSRM conditions.

For SRM tests, a mixture of vaporized water–methanol, with H_2_O/CH_3_OH molar ratios of 1.3:1, 2:1, and 4:1, and N_2_ carrier gas was cofed to the reactor using a mass flow controller (Bronkhorst High-Tech). The reaction gas mixture was composed of 80 vol % N_2_ and 20 vol % (CH_3_OH + H_2_O). The water–methanol solution was continuously dosed by a peristaltic pump. The gas hourly space velocity resulting from the total gas mixture was 40000 h^–1^. For OSRM tests, O_2_ was added to the reaction mixture SRM, using a mass flow controller (Bronkhorst High-Tech), with O_2_/H_2_O/CH_3_OH molar ratios of 0.1:1.3:1, 0.2:1.3:1, and 0.3:1.3:1. The other conditions were similar to those of SRM. The effluent gases were analyzed by online gas chromatography (3000A micro GC, Agilent Technologies, USA) equipped with three channels, using a molecular sieve, a Poraplot, QV-1 columns, and a TCD. The chromatograph was calibrated using known flow rates of pure gases (CO, CO_2_, and H_2_) with N_2_ as the internal standard. Other byproducts, such as methanol or ethylene, were not detected significantly. Methanol conversion and selectivity to products were determined as follows:
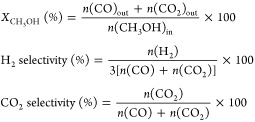
where *n*(CH_3_OH)_in_ is the flow rate (mol min^–1^) at the reactor inlet and *n*(CO)_out_, *n*(CO_2_)_out_, and *n*(H_2_)_out_ are the flow rates (mol min^–1^) of CO, CO_2_, and H_2_, respectively, at the reactor outlet.

## 3 Results and Discussion

### 3.1 Catalyst Characterization

**Figure 1:**
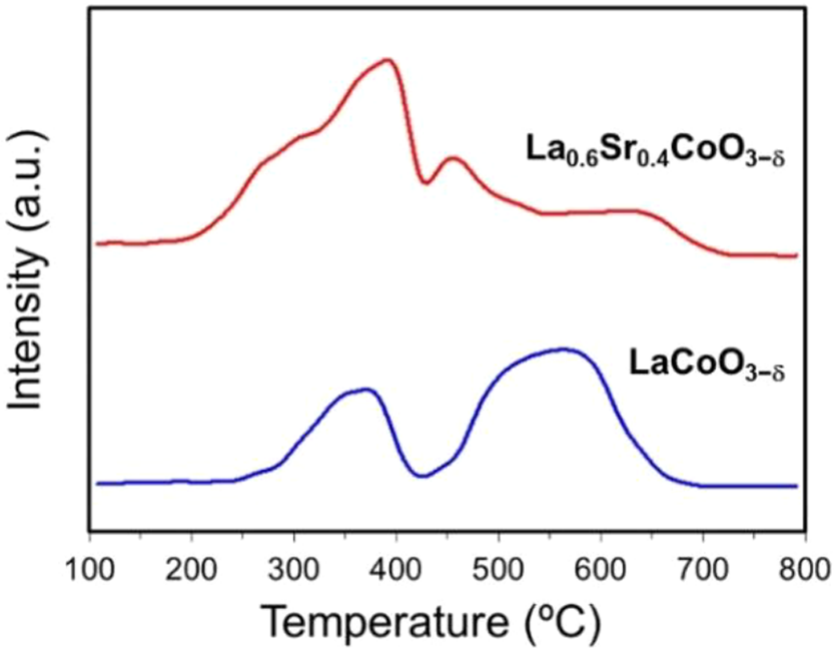
H_2_-TPR curves of the LSC and LCO perovskites.

Figure 1 shows the TPR results of LSC and LaCoO_3−δ_ (LCO). Co reduction in both catalyst precursors occurred in two steps. In the case of LCO, the initial reduction of Co^3+^ to Co^2+^ resulted between 300 and 420 °C with a peak centered at ∼380 °C, and the complete reduction of Co^2+^ to Co^0^ occurred at temperatures of 440–660 °C (maximum at ∼570 °C). Similar to the LCO perovskite, the Co of LSC was first reduced between 200 and 400 °C due to a one-electron reaction, which took place with two peaks at ∼280 and ∼380 °C. The second reduction step was a two-electron process in the 430–680 °C range, with two maxima centered at ∼440 and ∼640 °C. Compared to LCO, the TPR peaks of LSC were shifted toward lower temperatures. The Sr^2+^ doping in LSC led to a less stable structure, increasing the average oxidation state of Co^3+^ to Co^4+^ and/or the generation of oxygen vacancies.^53,54^ These TPR data suggested that the presence of Sr^2+^ in LCO-based perovskites should promote the reduction process and, consequently, the SRM of LSC at temperatures lower than those of LCO.

**Figure 2:**
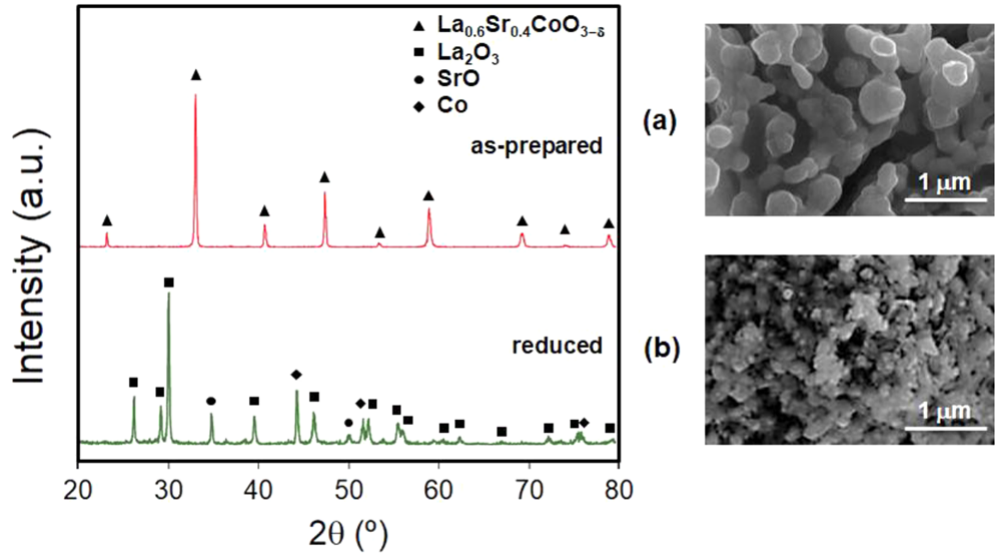
XRD patterns for LSC: (a) as-prepared and (b) after reduction in 5% H_2_/Ar at 650 °C for 1 h. Phases identified from JCPDS cards: cubic La_0.6_Sr_0.4_CoO_3−δ_ (JCPDS 48-0121), hexagonal La_2_O_3_ (JCPDS 05-0602), cubic SrO (JCPDS 48-1477), and cubic Co (JCPDS 15-0806).

**Figure 3:**
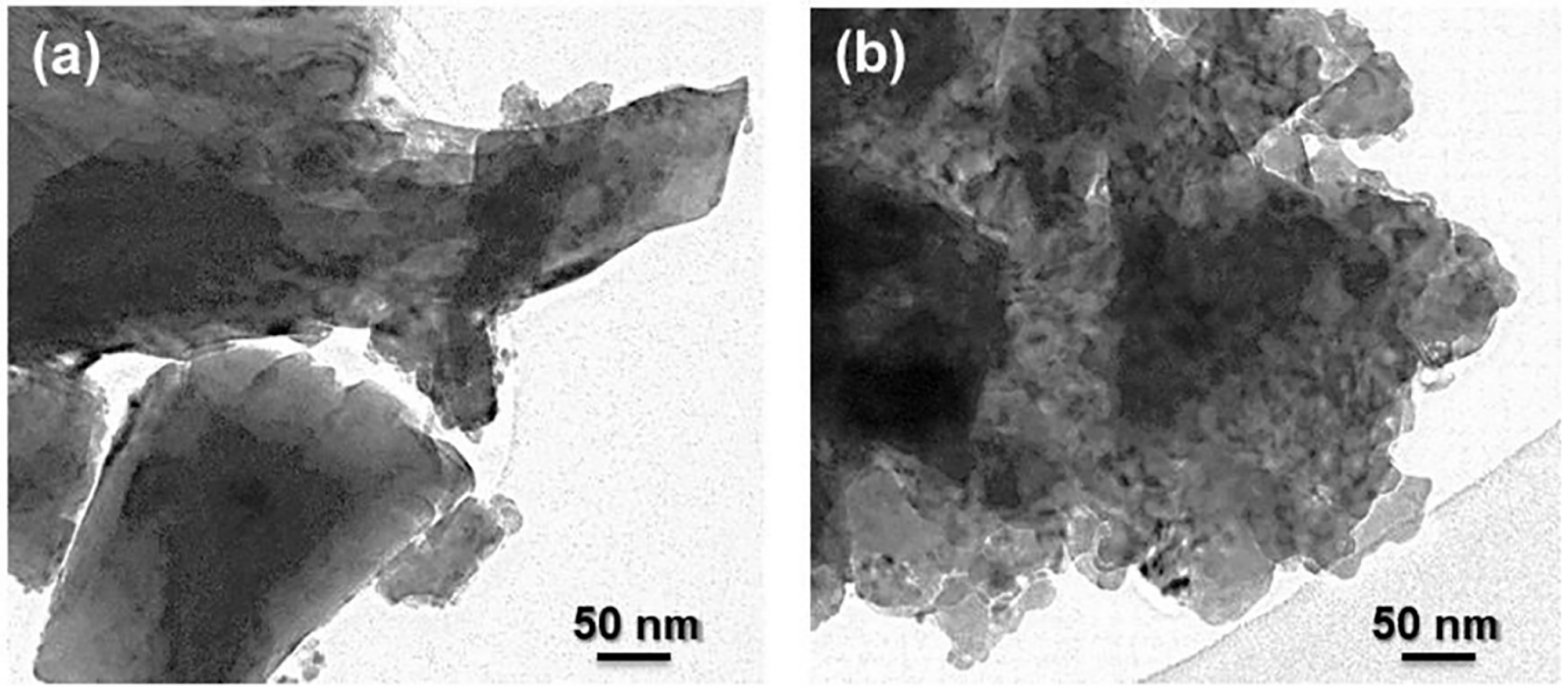
TEM images for LSC: (a) as-prepared and (b) after reduction in 5% H_2_/Ar at 650 °C for 1 h.

Figure 2 shows the XRD patterns and SEM images of LSC as-prepared and after a reductive thermal treatment at 650 °C for 1 h in 5 vol % H_2_/Ar. XRD analysis evidenced that as-prepared LSC presents the cubic perovskite structure, the only phase with a highly crystalline symmetry. After the reductive thermal treatment, XRD exhibited the formation of a mixture of phases composed of cubic Co, hexagonal La_2_O_3_, and cubic SrO. Therefore, due to the reduction reaction, the total decomposition of LSC generated the following process: La_0.6_Sr_0.4_CoO_3−δ_ → Co/La_2_O_3_/SrO. These results were in good agreement with the TPR data. The crystallite sizes were about 20–30 nm, as determined from the XRD data using Scherrer’s formula. This suggested that Co nanoparticles were highly dispersed on a La_2_O_3_ and SrO particles matrix. SEM and TEM micrographs of LSC showed that much larger particle dispersion and smaller particle size were achieved in the reduced state (Figures 2 and 3). The specific surface areas of the precursor increased from 6 m^2^ g^–1^ (as-prepared) to 56 m^2^ g^–1^ after the reduction process, which is in good agreement with the TEM images and XRD patterns. This strong increase in the specific surface area of the reduced precursor is expected due to the calcination conditions at temperatures as high as 900 °C to form the perovskite phase and the capacity of perovskite-type oxides for yielding an excellent metallic dispersion formed by one or several metals in a matrix of metal oxides.^18,55^

**Figure 4:**
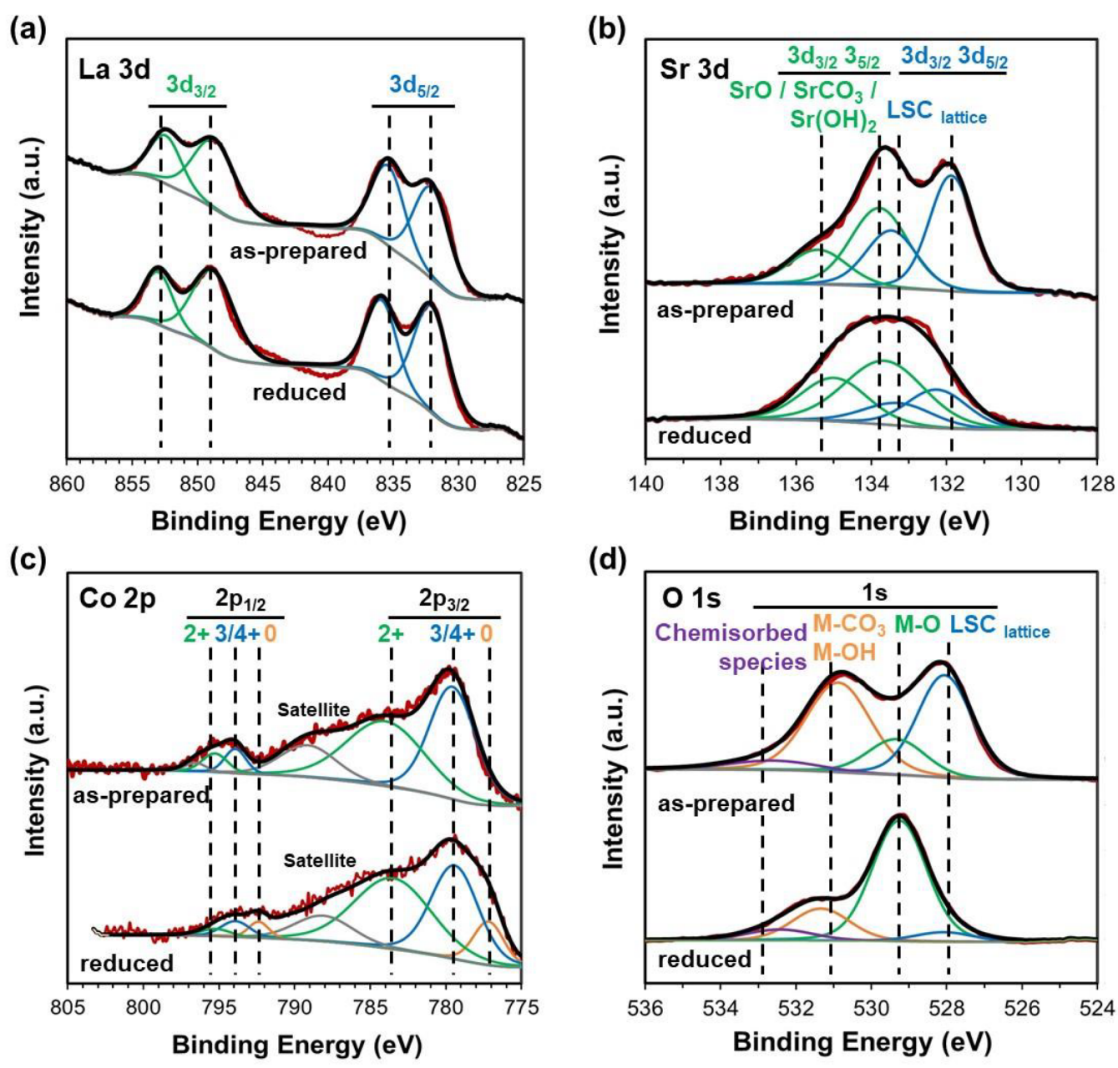
XPS spectra of (a) La 3d, (b) Sr 3d, (c) Co 2p, and (d) O 1s corresponding to LSC as-prepared and after the reduction process.

Figure 4 shows the XPS spectra of La 3d, Sr 3d, Co 2p, and O 1s corresponding to as-prepared LSC and after the reduction of LSC, which were obtained in an ultrahigh-vacuum chamber. Additionally, surface compositions of the samples derived from XPS measurements are listed in Table 1. The La 3d level was characterized by a double peak for each spin–orbit component (Figure 4a). The positions and shapes of the La 3d peaks for both samples were similar to those reported in the literature for LSC.^30,56^ In the La 3d signal, the double contribution of each spin–orbit is characteristic of La^3+^ and so is a fingerprint of the oxidation state. The samples exhibited different splittings (around 0.4 eV) in the La XPS peaks, 3.5 and 4.0 eV for the as-prepared and reduced samples, respectively. This is related to the chemical states present in each sample because the strong electron orbital–spin angular momentum interactions cause a splitting of the La XPS peaks. In the as-prepared sample, the splitting is close to that of La_2_(CO)_3_, in concordance with the Sr XPS results. After reduction, the splitting increased toward the value for lanthanum oxide, indicating that the carbonate amount decreased, but it is not pure lanthanum oxide bonding. The results are consistent with a mixture of La_2_O_3_, La_2_(CO)_3_, and La(OH)_3_. Figure 4b exhibits the Sr 3d_5/2_ region (∼132 eV) characteristic of Sr^2+^ in the perovskite phase (LSC lattice). In addition, the Sr 3d_3/2_ peak (∼133–134 eV) indicates the formation of SrCO_3_^56^ (∼133 eV), and the contribution at higher binding energy (∼134 eV) suggested the presence of SrO.^26^ The Sr 3d spectral comparison for the as-prepared and reduced LSC evidenced the significant intensity of the Sr 3d peak in SrCO_3_ after LSC reduction, which was associated with the Sr tendency to interact with the atmospheric CO_2_.^56^ For the as-prepared LSC in Figure 4c, the high intensities of Co 2p_1/2_ and Co 2p_3/2_ at binding energies of ∼779 and ∼783 eV and ∼794 and ∼795 eV, respectively, indicated that the Co was mainly in the states of Co^3+^/Co^4+^ and Co^2+^, respectively.^57,58^ As observed by other researchers for LSC,^59^ we note that Co^3+^ and Co^4+^ were indistinguishable and could not be quantified from the main peaks of the Co 2p XPS spectra, while both states are expected to exist on/in LSC at high temperatures. After the reduction treatment, both Co 2p peaks were wider than the as-prepared LSC due to the significant presence of metallic Co at ∼778 and ∼793 eV, respectively, and the decrease of the Co^3+^/Co^4+^ peak. In addition, the surface amount of Co after the reduction catalyst became higher than that of the as-prepared one, evidencing that the Co species got out on the surface. Figure 4d shows the O 1s region with four peaks at ∼528, ∼529, ∼531–532, and ∼533 eV attributed to lattice O in the perovskite, M–O (M = La, Co, Sr) in pure oxides, hydroxyl groups (∼531 eV), and carbonates species (∼532 eV), and chemisorbed water or O-containing species, respectively.^33,46,60^ After the reduction, the O 1s atom within the binding energy range of the LSC lattice was strongly decreased, suggesting a loss of O content at the surface of the reduced catalyst. An O loss at the surface of the reduced precursor was quantified from deconvolution of the fitted spectrum curves (Table 1). In addition, a certain segregation of the A site cation, especially in Sr, on the surface was observed in as-prepared and reduced precursors, which is in good agreement with previous works.^33,46,61^

**Table 1:** Nominal and XPS Compositions of LSC As-prepared and after the Reduction Process

		La	Sr	Co	O
Nominal (atomic %)		10	7	17	66
XPS (atomic %)	As-prepared	11	10	16	64
	After reduction	17	18	20	45

The characterization results suggest that a reductive pretreatment of LSC in 5 vol % H_2_/Ar at 650 °C for 1 h was beneficial to achieving highly dispersed metallic Co nanoparticles on a metal oxide matrix. Consequently, it was proposed to start the catalytic tests under SRM and OSRM with a totally reduced LSC catalyst precursor.

### 3.2 Catalytic Performance

#### 3.2.1 Effect of the Oxidation State of the Catalyst

**Figure 5:**
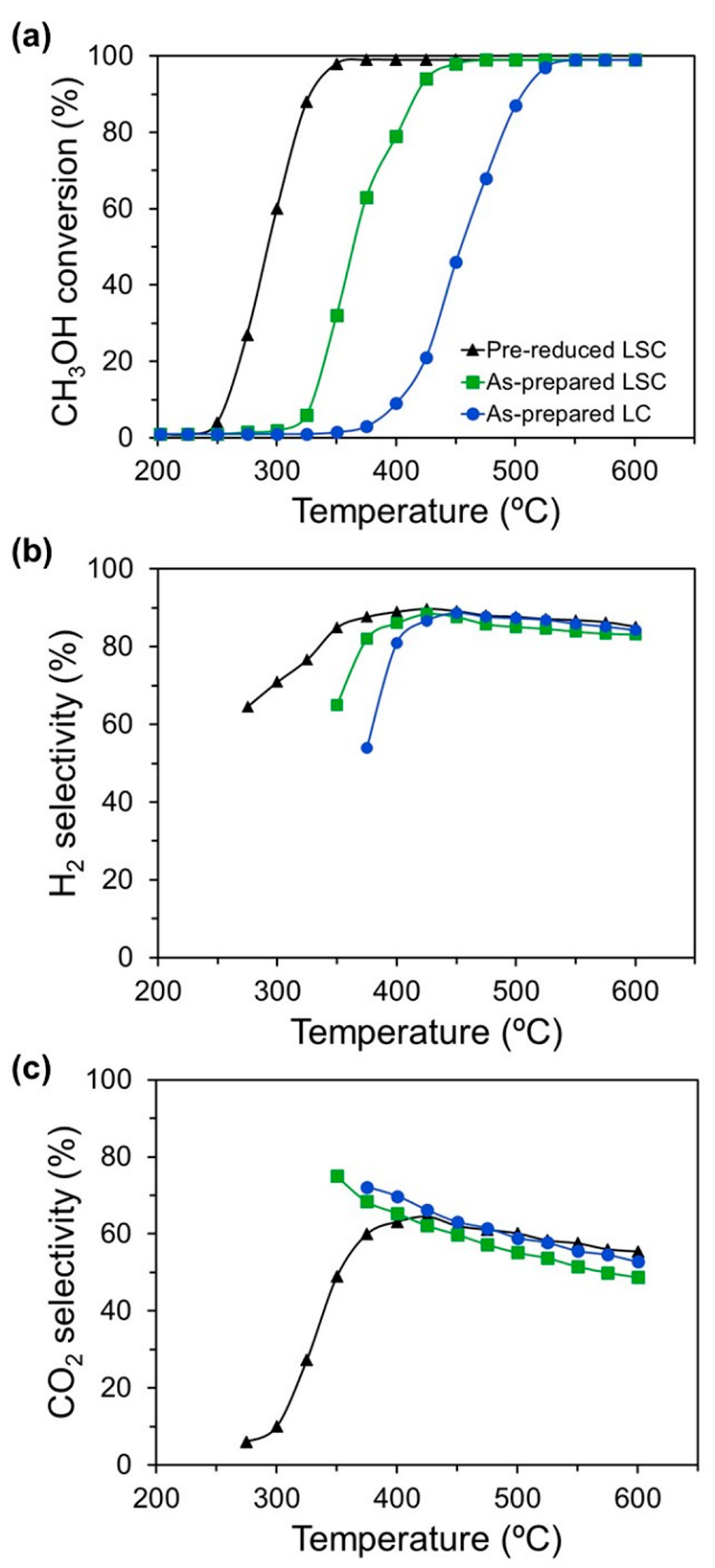
Effect of reductive thermal pretreatment in LSC on (a) CH_3_OH conversion, (b) H_2_ selectivity, and (c) CO_2_ selectivity at a H_2_O/CH_3_OH ratio of 1.3. The catalytic properties obtained for as-prepared LCO are reported for a comparison with those of as-prepared LSC.

Because the catalytic properties depend on the initial oxidation state of the catalyst, the effect of reductive thermal pretreatment of the catalyst precursor on the CH_3_OH conversion and product selectivity was investigated. Figure 5 shows the CH_3_OH conversion and CO_2_ and H_2_ selectivity under the SRM reaction, for an as-prepared LSC and after a reductive pretreatment in 5 vol % H_2_/Ar at 650 °C for 1 h. In addition, the catalytic properties obtained for an as-prepared LCO were also reported for comparison with the as-prepared LSC. As shown in Figure 5a, the significant CH_3_OH conversion of as-prepared LSC started at ∼300 °C and gradually enhanced with an increase of the temperature, and complete conversion was reached around 400 °C. In contrast, the catalytic activity of LSC after a reductive pretreatment at 650 °C remarkably enhanced at a lower temperature, as the CH_3_OH conversion started around 250 °C, and the total conversion was completed at ∼350 °C. In the temperature range of 275–425 °C, the CO_2_ and H_2_ selectivity progressively increased to about 65% and 90%, respectively (Figure 5b). Above 425 °C, the selectivity of CO_2_ and H_2_ dropped with an increase in the temperature, exhibiting a similar tendency for both as-prepared and pretreated LSC. These results confirmed that the previous state of the catalyst governed the catalytic activity at low temperature. The low CO_2_ and H_2_ selectivity values at low temperatures indicated the importance of the MD reaction, which decreased with an increase in the temperature to reach a maximum value at ∼425 °C. Above ∼425 °C, RWGS contributed to diminishing the CO_2_ and H_2_ selectivity, evidencing the critical role of thermodynamics in the SRM reaction over this catalyst. At this point, as reported in previous works,^18^ surface segregation of Co as Co^0^ induced by the reductive environmental conditions is a fundamental step for active species activation. The role of reductive thermal pretreatment of the catalyst precursor on the activation of active species at the microscopic level will be discussed in the next section on catalyst characterization under SRM and OSRM.

On the other hand, the Sr influence on the SRM performance of LSC was compared to that of undoped LCO. As shown in Figure 5a, the CH_3_OH conversion of as-prepared LCO started above ∼350 °C, and the total conversion was completed at ∼550 °C. Thus, the partial substitution of La by Sr reduced the temperature at which the catalyst started the SRM. These results were in good agreement with the TPR experiments, in which the TPR peaks of LSC were shifted toward lower temperatures concerning those of LCO. The values and trends of the CO_2_ and H_2_ selectivity for both LCO and LSC were close because the active phase exhibited similar behavior for both catalyst precursors (Figure 5b,c).

#### 3.2.2 Effect of the Feed Gas Composition

**Figure 6:**
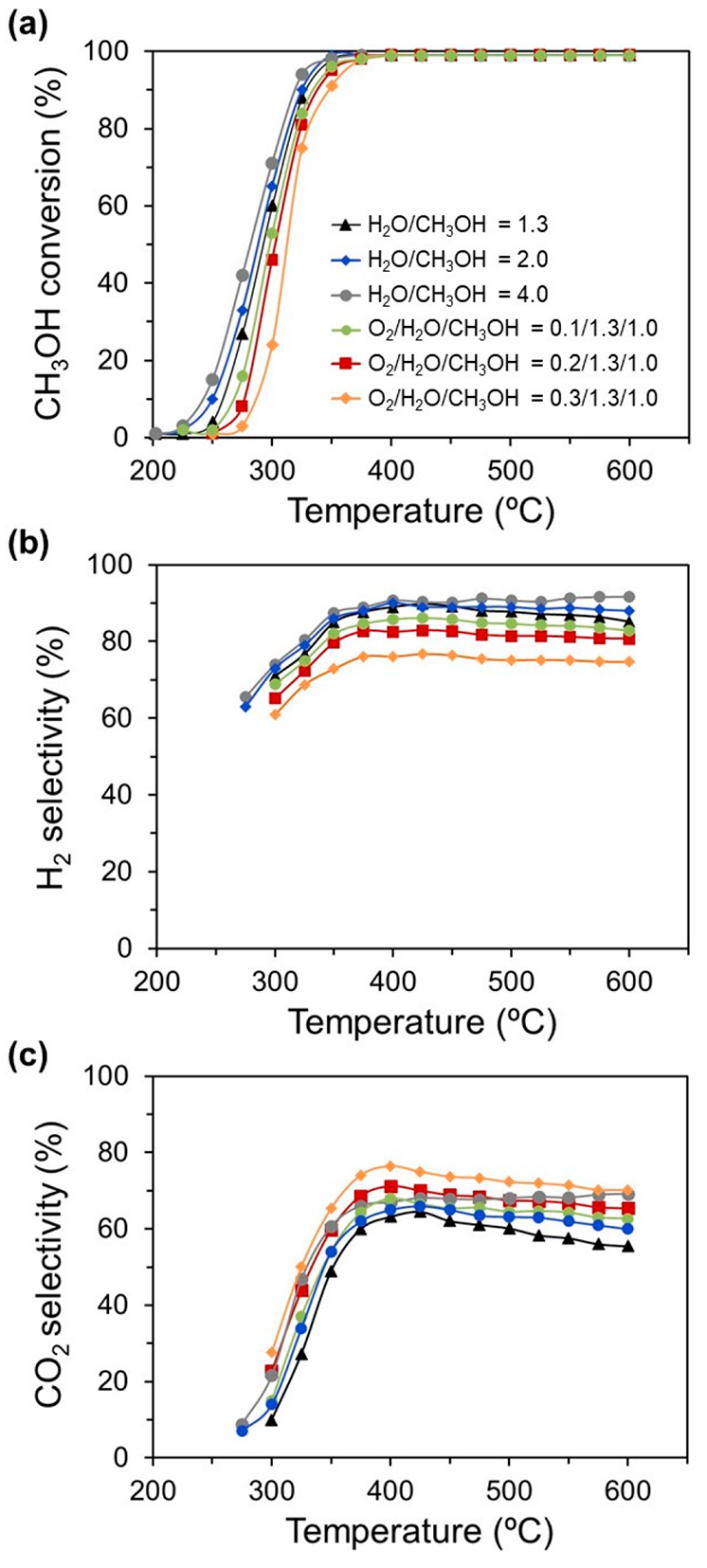
Effect of the feed composition on the (a) CH_3_OH conversion, (b) H_2_ selectivity, and (c) CO_2_ selectivity after a reductive pretreatment at 600 °C of LSC.

The feed gas composition in terms of the O_2_/H_2_O/CH_3_OH ratio was another important reaction variable that could strongly influence the catalytic activity. As shown in Figure 6, the increase of the H_2_O/CH_3_OH ratio from 1.3 to 4.0, under SRM conditions, led to a slight improvement of both the CH_3_OH conversion and selectivity of CO_2_ and H_2_ between 250 and 425 °C. Above ∼400 °C, a progressive drop of the CO_2_ and H_2_ selectivity with the temperature increase was observed for the H_2_O/CH_3_OH ratio of 1.3, which was associated with the RWGS effect. In contrast, the feed gas compositions with a larger excess of water exhibited a slight increase of both CO_2_ and H_2_. This behavior was related to the equilibrium of the WGS, which was thermodynamically predominant above 400 °C for high H_2_O/CH_3_OH ratios, thus contributing to the formation of CO_2_ and H_2_ at the expense of CO.

On the other hand, the effect of the H_2_O/CH_3_OH ratio was also significant to the stability of the catalyst. While no remarkable deactivation of the catalyst activity was observed using an H_2_O/CH_3_OH ratio of 4.0, a progressive activity loss with the time-on-stream reaction was detected for a ratio of 1.3. This was attributed to the carbon deposition because the carbon present in the catalyst was observed by SEM–EDS analysis. Therefore, the feed gas composition with the most water excess (H_2_O/CH_3_OH = 4.0) yielded better CH_3_OH conversion and CO_2_ and H_2_ selectivity and stability in the whole temperature range than that close to the stoichiometric one (H_2_O/CH_3_OH = 1.3).

Finally, the effect of the addition of O_2_ to SRM (O_2_/H_2_O/CH_3_OH = 0.1–0.3/1.3/1.0) on the catalytic performance was studied. As shown in Figure 6, CH_3_OH conversion under OSRM, compared to SRM, revealed a slight shift in the temperature at which the catalyst started to be active. This was attributed to the presence of O_2_ because it could slightly deactivate the catalyst surface at low methanol conversions without H_2_ and CO production. Then, the Co surface could be reoxidized to cobalt oxide, deactivating the catalyst for methanol reforming. At higher temperatures, the gas mixture becomes reductive again, and cobalt oxide is transformed into metallic Co because the combustion consumes O_2_. As a consequence, CH_3_OH conversion and product selectivity presented a similar trend in both processes. As expected, while H_2_ generation decreased with the addition of O_2_, CO_2_ increased. For instance, for an initial molar ratio of O_2_/H_2_O/CH_3_OH = 0.2/1.3/1.0, the corresponding H_2_O/CH_3_OH ratio after complete O_2_ consumption would be close to 2. Thus, OSRM was a way of increasing the H_2_O/CH_3_OH ratio and decreasing CO_2_ production by hindering RWGS. In addition, no significant deactivation of the catalyst activity with the time-on-stream reaction was observed in OSRM. Therefore, the addition of O_2_ to the SRM reaction could be an effective way of avoiding carbon deposition and increasing the CO_2_ selectivity, consequently decreasing CO generation.

### 3.3 Catalyst Characterization after SRM and OSRM

**Figure 7:**
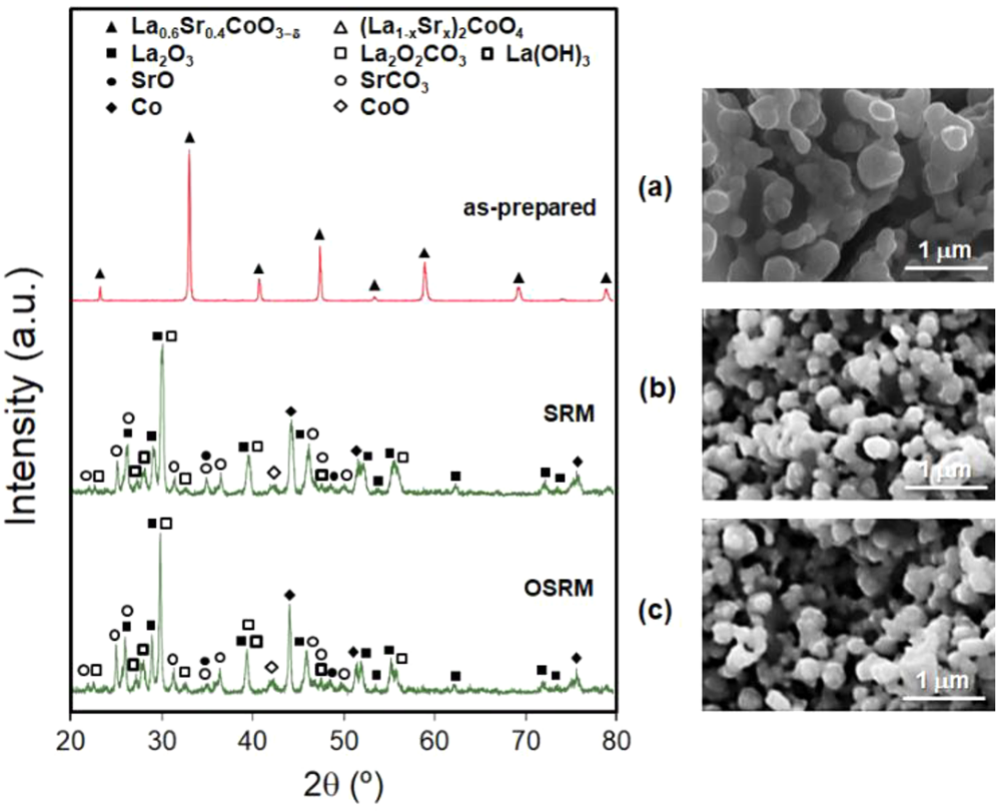
XRD patterns and SEM images for LSC: (a) as-prepared and after prereduction in 5% H_2_/Ar at 600 °C for 1 h, operating under (b) SRM and (c) OSRM at 600 °C for 1 h, and quenching in a N_2_ atmosphere. Phases identified from JCPDS cards were as follows: cubic La_0.6_Sr_0.4_CoO_3−δ_ (JCPDS 48-0121), hexagonal La_2_O_3_ (JCPDS 05-0602), hexagonal La(OH)_3_ (JCPDS 36-1481), monoclinic La_2_O_2_CO_3_ (JCPDS 48-1113), cubic SrO (JCPDS 48-1477), orthorhombic SrCO_3_ (JCPDS 05-0418), cubic Co (JCPDS 15-0806), and cubic CoO (JCPDS 71-1178).

In order to study the evolution of the phases formed at the catalyst under SRM and OSRM, all samples were characterized by using XRD. Figure 7 shows the XRD patterns of the as-prepared catalyst and after testing under SRM and OSRM conditions at 600 °C for 1 h and quenched in a N_2_ atmosphere. After the SRM and OSRM reactions, the characteristic diffraction peaks of hexagonal La_2_O_3_, hexagonal La(OH)_3_, monoclinic La_2_O_2_CO_3_, cubic SrO, orthorhombic SrCO_3_, cubic Co^0^, and cubic CoO were detected, and no peaks of LSC were observed (Figure 7b,c). This evidenced that Co ions were reduced from the perovskite lattice to Co^0^ nanoparticles, which were highly dispersed on a matrix of lanthanum and strontium oxides (Co/La_2_O_3_/SrO). The Co^0^ crystallite sizes determined from the XRD data with Scherrer’s formula were 20–30 nm. In addition, other species such as La_2_O_2_CO_3_, La(OH)_3_, SrCO_3_, and CoO were also formed under the SRM and OSRM conditions. The reactions of La_2_O_3_ with H_2_O and SrO with CO_2_ as reaction products generated La(OH)_3_ and SrCO_3_ (eqs 5 and 6, respectively).
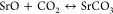
5
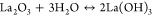
6

As reported in previous works,^62−64^ the presence of La_2_O_2_CO_3_ was especially interesting because lanthanum oxycarbonate species could react with surface carbon in their vicinity, thus cleaning the Co surface of the carbon deposits. In this way, the adsorbed CO_2_ on La_2_O_3_ formed La_2_O_2_CO_3_ (eq 7), which could react with neighboring active carbonaceous intermediate species to produce CO and regenerate La_2_O_3_, completing the cycle between La_2_O_2_CO_3_ and La_2_O_3_ (eq 8):
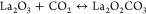
7
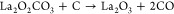
8

This mechanism described the good stability of the catalysts based on M/La_2_O_3_ (M = Co, Ni, etc.) under reforming reaction conditions because active metal particles were decorated with lanthanum oxide species originating from the support. Regeneration of the active sites took place in the vicinity of lanthanum oxide particles with an intermediate formation of La_2_O_2_CO_3_. However, this mechanism in the LSC precursor showed limited efficiency in severe SRM conditions because catalyst deactivation was observed for nearly stoichiometric feed ratios (H_2_O/CH_3_OH = 1.3). Thus, the good catalyst stability exhibited under SRM for an H_2_O/CH_3_OH ratio of 4, and also under OSRM, could be attributed to the influence of lanthanum oxycarbonate. As shown in Figure 7, the formation of lanthanum oxycarbonate was favorable in a high excess of water and an addition of O_2_, which reduced the potential catalyst deactivation caused by carbon deposition. SEM micrographs confirmed that both the dispersion and size of the particles after reduction of the catalyst precursor were more significant than those of the as-prepared ones (Figure 7). Similarly, the specific surface area of the as-prepared catalyst precursor (6 m^2^ g^–1^) was strongly increased to 25 and 28 m^2^ g^–1^ after testing and quenching in N_2_ under SRM and OSRM, respectively. The superior catalytic performance of OSRM compared to SRM was attributed to the higher surface area, higher metal dispersion, and smaller size of the Co^0^ nanoparticles.

### 3.4 Catalytic Performance in Redox Cyclic Tests

**Figure 8:**
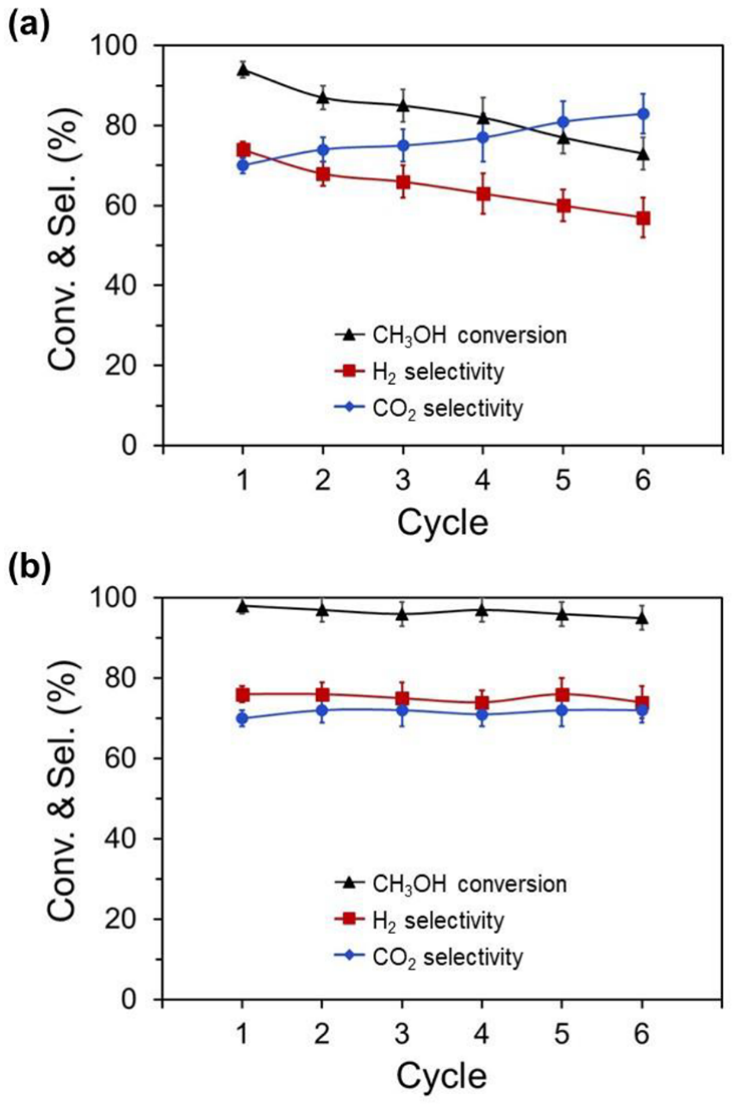
CH_3_OH conversion and selectivity of H_2_ and CO_2_ in the redox cyclic tests operating under OSRM reaction conditions at 600 °C and O_2_/H_2_O/CH_3_OH = 0.1–0.3/1.3/1.0 and subsequently (a) slow cooling to RT under OSRM and (b) reoxidized in air at 900 °C and then slow cooling to RT in air.

As mentioned above, the best resistance of the catalyst to deactivation due to carbon deposition was under OSRM reaction conditions (O_2_/H_2_O/CH_3_OH = 0.1–0.3/1.3/1.0). Therefore, these reaction conditions were selected to evaluate the effect of redox cycles on the catalytic activity and *in situ* self-regeneration of the catalyst, thus completely repeating several times the process of reduction and oxidation for the same sample. Figure 8 shows the CH_3_OH conversion and selectivity of CO_2_ and H_2_ of the precursor focused on two different cyclic tests: (a) operating under OSRM at 600 °C for 1 h and slow cooling to RT under OSRM (Figure 8a) and (b) operating under OSRM and reoxidized in air at 900 °C and then slow cooling to RT in air (Figure 8b). In the redox cyclic tests under OSRM, both the CH_3_OH conversion and H_2_ selectivity were strongly decreased with an increase in the redox cycles. In contrast, the CH_3_OH conversion and selectivity of CO_2_ and H_2_ during the six cyclic tests with a specific thermal treatment remained almost constant, indicating that the conditions of the reoxidation process play a remarkable role in the performance and stability of the catalyst in successive redox processes.

### 3.5 Catalyst Characterization after Redox Cyclic Tests

**Figure 9:**
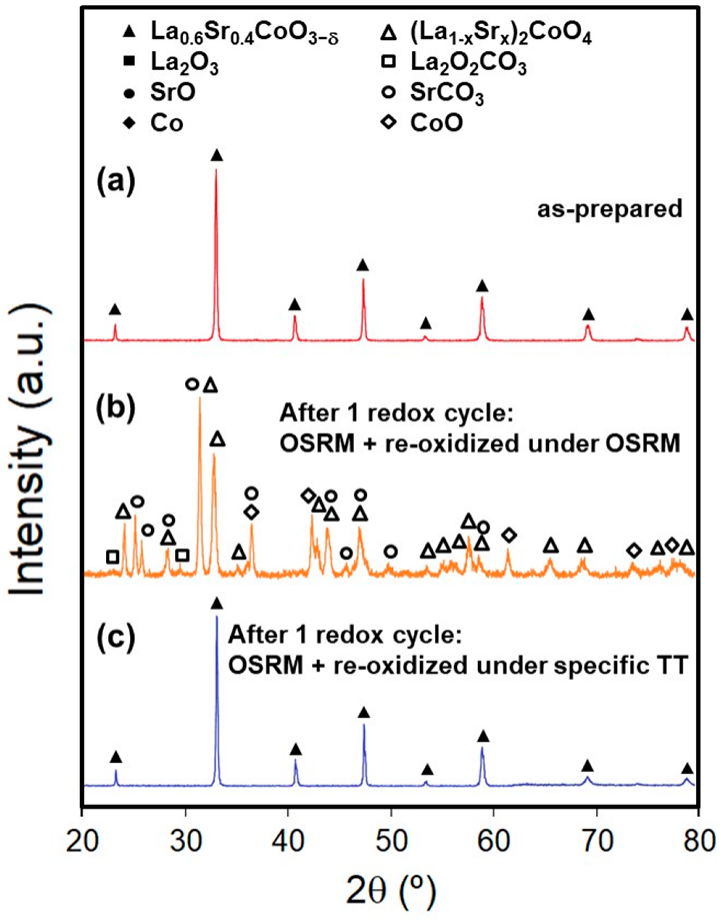
XRD patterns for LSC: (a) as-prepared; (b) after one redox cycle of prereducing in 5% H_2_/Ar at 650 °C for 1 h, operating under OSRM at 600 °C for 1 h, and slow cooling to RT under OSRM; (c) after one redox cycle of prereducing in 5% H_2_/Ar at 650 °C for 1 h, operating under OSRM at 600 °C for 1 h, and reoxidized under specific thermal treatment of regeneration in air at 900 °C for 12 h and in pure O_2_ at 300 °C for 72 h. Phases identified from JCPDS cards were as follows: cubic La_0.6_Sr_0.4_CoO_3−δ_ (JCPDS 48-0121), tetragonal (La_1–*x*_Sr_*x*_)_2_CoO_4_ (*x* = 0; JCPDS 34-1296), monoclinic La_2_O_2_CO_3_ (JCPDS 48-1113), orthorhombic SrCO_3_ (JCPDS 05-0418), and cubic CoO (JCPDS 71-1178).

**Figure 10:**
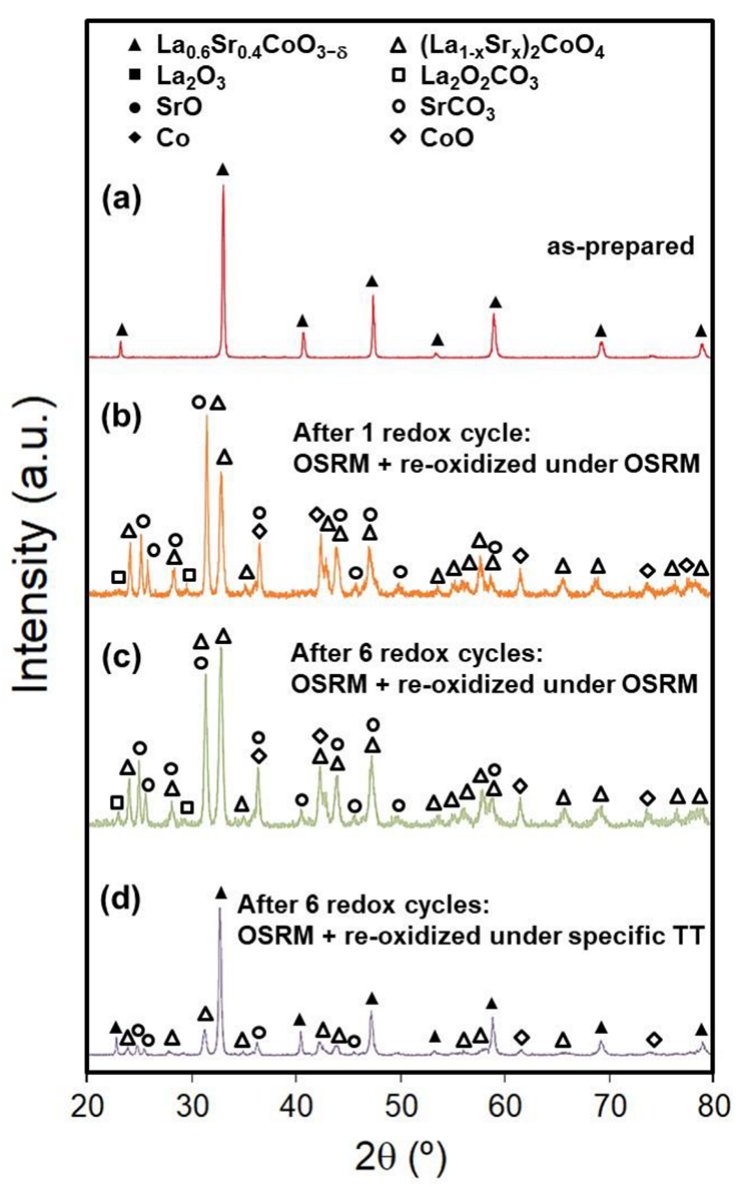
XRD patterns for LSC: (a) as-prepared; (b) after one redox cycle of prereducing in 5% H_2_/Ar at 650 °C for 1 h, operating under OSRM at 600 °C for 1 h, and slow cooling to RT under OSRM; (c) after six redox cycles, repeating 6 times similar to one cycle, and slow cooling to RT under OSRM; (d) after six cycles and regeneration in air at 900 °C for 12 h and in pure O_2_ at 300 °C for 72 h. Phases identified from JCPDS cards were as follows: cubic La_0.6_Sr_0.4_CoO_3−δ_ (JCPDS 48-0121), tetragonal (La_1–*x*_Sr_*x*_)_2_CoO_4_ (*x* = 0; JCPDS 34-1296), monoclinic La_2_O_2_CO_3_ (JCPDS 48-1113), orthorhombic SrCO_3_ (JCPDS 05-0418), and cubic CoO (JCPDS 71-1178).

XRD analyses were carried out to determine the evolution of phases in the LSC precursor after redox cyclic tests under OSRM and *in situ* self-regeneration of the catalyst (Figure 9). As shown in Figure 9, the XRD patterns after the first cycle exhibited the formation of the tetragonal phase [T′(La_1–*x*_Sr_*x*_)_2_CoO_4_] due to the reaction of La_2_O_3_, SrO, and Co favored by slow cooling during the cyclic tests. In addition, a small fraction of La, Sr, and Co remained as La_2_O_2_CO_3_, SrCO_3_, and CoO. Therefore, the self-regeneration process of the catalyst in a redox cycle was not completed under the OSRM reaction conditions. However, the XRD results evidenced that the used catalyst after one redox cycle could be regenerated entirely under a reoxidation thermal treatment in synthetic air at 900 °C for 12 h and finally in pure O_2_ at 300 °C for 72 h (Figure 9c). Complete regeneration of the used catalyst suggests that Co nanoparticles were highly dispersed on the matrix of lanthanum and strontium carbonates and oxides under OSRM, which could be interesting for the mitigation of Co coarsening and the deactivation of the catalyst in operation. In another series of experiments, degradation and regeneration were studied in successive redox cycles (Figure 10). After six redox cycles, the XRD patterns showed higher amounts of the same compounds ([T′(La_1–*x*_Sr_*x*_)_2_CoO_4_], La_2_O_2_CO_3_, SrCO_3_, and CoO) compared to the first cycle (Figure 10b,c). In this regard, the used catalyst after six redox cycles could not be regenerated entirely under a reoxidation thermal treatment in synthetic air at 900 °C for 12 h and finally in pure O_2_ at 300 °C for 72 h (Figure 10d). In this case, the catalyst presented small amounts of (La_1–*x*_Sr_*x*_)_2_CoO_4_, and the presence of SrCO_3_ and CoO was also detected; therefore, the irreversible segregation of Sr-rich species took place.

**Figure 11:**
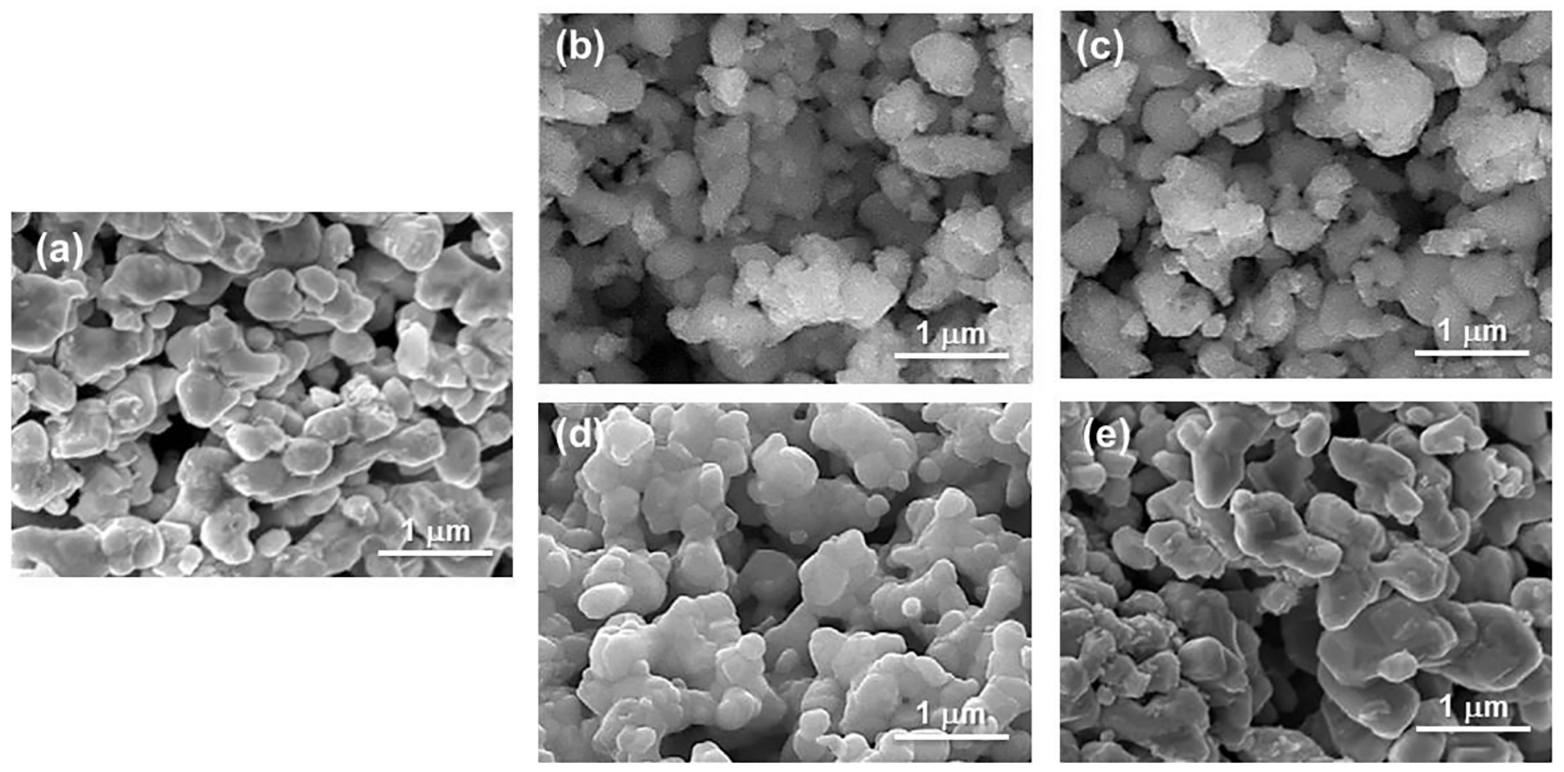
SEM images of LSC: (a) as-prepared; after (b) one and (c) six redox cycles, operating under OSRM at 600 °C for 1 h and slow cooling to RT under OSRM; after (d) one and (e) six redox cycles, operating under OSRM at 600 °C for 1 h and a regeneration thermal treatment in air at 900 °C for 12 h and in pure O_2_ at 300 °C for 72 h.

SEM images of the as-prepared catalyst and after redox cyclic tests and thermal treatment of regeneration are shown in Figure 11. After the first redox cycle, the used catalyst and slow cooling in OSRM presented slightly smaller particle sizes than the as-prepared and regenerated catalysts (Figure 11a,b,d). However, after the sixth cycle, it can be observed that both the used catalyst (slow cooling under OSRM) and the regenerated catalyst exhibited the grains bonded together slightly, evidencing a slight sintering after redox cycles at high temperature (Figure 11a,c,e). In addition, the morphology of the used catalysts after the first cycle (Figure 10b,d) exhibited more porosity and larger voids between particles compared to the six cycles (Figure 11c,e). The interconnected porosity and voids between the particles enable the diffusion of reactant and product gas into the solid particles. These observations in the microstructures are consistent with the values of the specific surface area because the used catalyst after the first cycle presented a higher specific surface area (12 m^2^ g^–1^) than the regenerated catalyst (7 m^2^ g^–1^) and the as-prepared one (6 m^2^ g^–1^). In contrast, the specific surface area significantly decreased during successive redox cycles, reaching values of 8 and 5 m^2^ g^–1^ (after six cycles) for the slow cooling and regenerated catalysts, respectively.

With the experimental observations discussed above, we can suggest how the different programs of the redox cycles (in terms of reoxidation and cooling from the operation temperature to RT) affect the regeneration of a catalyst. In the first program of the redox cycle, reoxidation of the catalyst with slow cooling under OSRM caused severe degradation of the catalytic activity in successive redox cycles. This was attributed to a remarkable segregation of phases [(La_1–*x*_Sr_*x*_)_2_CoO_4_, SrCO_3_, ...] with the accumulation of redox cycles in an irreversible way, of which the entire regeneration of the catalyst using a regeneration thermal treatment is difficult. As reported in previous studies of LSC for electrochemical applications such as SOFCs,^46,65^ the accumulation of Sr-rich species, particularly SrCO_3_, on the catalyst surface covered the catalytically active Co surface sites, thus very effectively decreasing the catalytic activity of LSC. In the second program of the redox cycle, the use of a specific regeneration treatment in air at 900 °C between consecutive cycles stopped degradation of the catalytic activity, which suggests that the regenerated LSC presented a surface quite similar to the original one.

## 4 Conclusions

SRM and OSRM over LSC perovskite, as a catalyst precursor, were studied to supply syngas enriched in H_2_ for SOFC applications. The SRM performance of the catalyst precursor strongly depended on the initial oxidation state of the catalyst, feed gas composition, and reaction temperature. CH_3_OH conversion of the catalyst with a reductive pretreatment at 650 °C was significantly enhanced at lower temperatures than the as-prepared one. Sr doping in LaCoO_3_-based perovskites facilitated the reduction process for the as-prepared catalyst precursor. Consequently, CH_3_OH conversion of LSC under SRM was increased at lower temperatures compared to undoped LaCoO_3_. MD and RWGS occurred simultaneously with SRM and OSRM. The SRM performance was also significantly affected by the feed gas composition. The feed composition with significant water excess (CH_3_OH/H_2_O = 4.0) yielded better CO_2_ and H_2_ selectivity (at temperatures of >400 °C) than that close to the stoichiometric one due to the increases of the WGS reaction. In addition, this reduced the potential catalyst deactivation caused by carbon deposition. In OSRM, adding O_2_ to SRM was an effective way of avoiding carbon deposition but at the expense of reducing the H_2_ selectivity and increasing the CO_2_ selectivity. The characterization results evidenced the stability of highly dispersed Co nanoparticles over a matrix composed of metal carbonates and oxides, mainly La_2_O_3_, La_2_O_2_CO_3_, SrO, and SrCO_3_. In addition, the formation of lanthanum oxycarbonate could also contribute to minimizing carbon deposition on the catalyst surface.

The identification of O loss and certain segregation of Sr-rich species on the reduced precursor surface by XPS evidenced a strong connection between the surface chemistry and catalytic activity. The reoxidation program in repetitive redox cycles played a crucial role in the regeneration of catalysts. A reoxidation with slow cooling under OSRM contributed to a severe degradation of the catalytic activity in successive redox cycles probably due to the accumulation of Sr-rich species (SrCO_3_) on the catalyst surface, thus covering the catalytically active Co surface sites. In contrast, the reoxidized catalyst in air at 900 °C and then slow cooling to RT between consecutive redox cycles stopped degradation of the catalytic activity. Furthermore, the catalyst precursor could be regenerated to the initial perovskite structure with a specific regeneration treatment (in air at 900 °C for 12 h and in pure O_2_ at 300 °C for 72 h). The authors recognize that these results do not demonstrate long-term cycling stability of LSC; however, it can be an effective approach to mitigating the degradation of LSC, enabling its practical application in catalysis but also in other systems such as SOECs for renewable energy conversion and storage.
